# Modulation of Phytochemicals and Essential Trace Elements in Fruits of Different Tomato Cultivars by the Endophytic Fungus *Penicillium pinophilum* EU0013

**DOI:** 10.1264/jsme2.ME22026

**Published:** 2022-09-14

**Authors:** Sarah Remi Ibiang, Kazunori Sakamoto

**Affiliations:** 1 Graduate School of Horticulture, Chiba University, 648 Matsudo, Matsudo, Chiba 271–8510 Japan

**Keywords:** plant-microbe interaction, food quality, bio-fortification, phytohormones, tomato

## Abstract

The present study investigated the effects of the endophytic fungus, *Penicillium pinophilum* EU0013 on fruit phytochemical indices and essential trace elements in five tomato cultivars. In a completely randomized design, inoculated and uninoculated seedlings of tomato cultivars (Momotaro, Rodeo, Anaya, Reika, and Cherry) were raised for sixteen weeks in a greenhouse. Fruit fresh weights and root colonization by *P. pinophilum* were significantly higher in the Rodeo cultivar than in the other cultivars tested. Significant effects of the cultivar, inoculation, and interaction on fruit dry weights were‍ ‍observed with higher values in Anaya inoculated with *P. pinophilum*. Cultivar and inoculation effects were significant for ascorbic acid and soluble sugars in four cultivars, with increases being observed due to the *P. pinophilum* inoculation. Lycopene levels increased in Rodeo and decreased in Anaya, while β-carotene levels increased in four cultivars due to the inoculation. Manganese concentrations were significantly increased in Cherry, while iron concentrations were increased in Reika and Cherry. Increases due to the inoculation were observed for gibberellic acids (GA_1_ and GA_4_) in Reika and Anaya, whereas decreases were detected in Cherry and Rodeo. Similar results were obtained for abscisic acids (ABA) with increases in Reika and Anaya due to the inoculation. *P. pinophilum* EU0013 demonstrated the ability to improve the nutritive value of tomato fruits via modulations to phytochemicals in addition to increases in Mn and Fe concentrations, particularly in Cherry and Rodeo. Cultivar responses to the *P. pinophilum* inoculation are a factor that need to be considered for its use in increasing fruit quality indices in tomato.

Tomato (*Solanum lycopersicum* L.) is a popular and highly consumed fruit/vegetable worldwide. It is considered to be one of the most economically important crops worldwide and is a rich source of minerals, vitamins, organic acids, essential amino acids, and dietary fibers, which are vital for the general health of humans by minimizing the risk of diseases and other medical conditions (Wargovich, 2000; Oguntibeju, 2013). The nutritive value of tomato fruits, such as color, flavor, and taste, mainly depends on lycopene, β-carotene, ascorbic acid, gibberellic acids (GA), sugars, indole acetic acids (IAA), abscisic acid (ABA), and essential trace elements (Ilahy *et al.*, 2009). Lycopene and β-carotene are among the most important carotenoids in tomato and function as antioxidants. However, their accumulation in fruits depends on the cultivar/genotype, ripening stage, and environmental factors (Tilahun *et al.*, 2017). Environmental factors include the prevailing abiotic and biotic conditions impacting plants, which interact with genetic factors, such as the cultivar type, to shape the plant nutrient physiology.

Food quality and human health are intertwined. From essential trace elements, such as iron (Fe), zinc (Zn), and manganese (Mn), to antioxidative phytochemicals, including polyphenols, β-carotene, and ascorbic acids, the nutrient quality of foods needs to be assessed and improved for many individuals who may be affected by insufficient intake via the diet (FAO, 2020). There is also an increased recognition of the value of natural over synthetic antioxidants in our diets for the modulation of oxidative stress stemming from injury and metabolic disorders (Mahmood *et al.*, 2021). Microbial biofortification is a potentially attractive plant-microbe interaction strategy for improving the nutrient quality of foods because the bacterial and fungal modulation of phytochemicals in host plants and the rhizosphere may lead to higher levels of polyphenols, organic acids, sugars, and micronutrients (Khan *et al.*, 2009; Ibiang *et al.*, 2018). Endophytes are microorganisms that inhabit the internal parts of plants for at least part of their life cycle without causing disease and include *Penicillium* sp., *Colletotrichum tofieldiae* (Hiruma *et al.*, 2016), and *Acremonium* sp. (Khan *et al.*, 2021). *Penicillium* sp. are popular endophytes for their secretion of bioactive compounds and roles as biocontrol agents against plant diseases (Guijarro *et al.*, 2017). The endophytic fungus *Penicillium pinophilum* EU0013, isolated from eucalyptus roots (Teshima and Sakamoto, 2006), has been shown to improve seed germination, plant growth, and tolerance to *Fusarium* wilt in tomato (Alam *et al.*, 2011). Its abilities to solubilize phosphate, produce organic acids and siderophores *in vitro*, and improve tolerance to *Verticillium* wilt in tomato have recently been demonstrated (Ibiang *et al.*, 2020, 2021). It is now being considered as a candidate for more widespread deployment; however, its impact on fruit quality indices in tomato remain unclear. Therefore, the present study investigated the effects of *P. pinophilum* EU0013 inoculation on fruit phytochemical indices and essential trace elements, namely Zn, Fe, and Mn, in five tomato cultivars obtained from Nigeria and Japan. Since *P. pinophilum* was originally isolated in Japan, we broadened the cultivar domicile by utilizing two from Nigeria (tropical region) and three from Japan (temperate region).

## Materials and Methods

### Soil

The soil utilized was a mixture of river sand and commercial soil (Premium soil, Setoharakaen) with pH 7.4, EC of 75.8‍ ‍mS‍ ‍m^–1^, available Zn of 25.4‍ ‍μg‍ ‍g^–1^ soil, Fe of 16.1‍ ‍μg‍ ‍g^–1^ soil, Mn of 11.9‍ ‍μg‍ ‍g^–1^ soil, and Cu of 0.3‍ ‍μg‍ ‍g^–1^ soil. River sand was sieved with a 2-mm mesh and both soils were mixed (1:1 v/v), autoclaved at 121°C for 2 h, and then allowed to cool for 2‍ ‍d before use.

### Plant

Five tomato (*Solanum lycopersicum* L.) cultivars (Momotaro, Rodeo, Anaya, Reika, and Cherry) were examined in the present study. Anaya and Rodeo (supplied by AGRITROPIC, Nigeria) are planted by farmers in Nigeria, while Reika, Cherry, and Momotaro (supplied by Takii Seed) are cultivated in Japan. Tomato seeds were sterilized in 70% ethanol for 5‍ ‍min and 33.3% sodium hypochlorite solution for 15‍ ‍min and then rinsed repeatedly in sterile distilled water before seeding in a growth chamber to obtain seedlings. The conditions in the growth chamber were as follows: 140‍ ‍μmol m^–2^ s^–1^ fluorescent light (25°C, 14 h) and dark (18°C, 10‍ ‍h). Seedlings were raised on vermiculite moistened with half-strength Hoagland solution for four weeks before being transplanted to potted soils.

### Penicillium inoculation

*P. pinophilum* EU0013 (available from the National Institute of Technology and Evaluation, NBC accession number 100411) was obtained from actively growing margins of 7- to 10-d-old potato dextrose agar (PDA) culture medium. A wheat bran substrate-based inoculum was prepared for the *Penicillium* inoculation. The inoculum was prepared as described by Chandanie *et al.* (2006), but with an incubation temperature of 25°C. After a 10-d incubation, completely colonized wheat was blended, and 0.5‍ ‍g (3.6×10^3^ spores) was added to the middle of the designated pots before seedling transplant, while non-penicillium pots received no inoculum (Ibiang *et al.*, 2020).

### Experimental design and plant cultivation

The experiment was laid out in a randomized 5×2 factorial representing cultivars and inoculation treatments. Regarding the inoculation, uninoculated control (EU0013–) and inoculated (EU0013+) groups were set up for each cultivar, with three replicates (*n*=3), giving 30 pots. Plant cultivation was performed between May and August 2020 in a greenhouse for 16‍ ‍weeks. All planted pots were equally fertilized with full-strength Hoagland solution applied (100‍ ‍mL pot^–1^) once every two weeks until harvesting, while regular watering was conducted with tap water.

### Data collection

All fruits produced by the respective plants were harvested after 16‍ ‍weeks, weighed, and sorted into green, breaker, turning, pink, light red, and red to cover all of the ripening stages of tomato (de Jong *et al.*, 2009). Light red and red fruits (stages 5 and 6) were more abundant than the others and were utilized in fruit phytochemical and essential element ana­lyses. Selected fruits were longitudinally sectioned into four equal parts, and one randomly selected portion for the phytochemical ana­lysis was wrapped with aluminum foil and submerged in liquid nitrogen before being stored at –80°C. Leftover fruit tissues were placed in an oven for drying at 105°C until a constant weight, and dry fruit weights were recorded for each plant. Roots were obtained and washed under running tap water, and a portion was subtracted to assess endophyte root colonization.

### Root colonization by *Penicillium*

Root samples were washed thoroughly with running tap water and washed again with sterile water before use. Ten root segments of 0.5‍ ‍cm were cut and placed on PDA media in Petri dishes amended with a penicillin antibiotic (200‍ ‍mg L^–1^) to suppress the growth of other ubiquitous microorganisms. Petri dishes were placed in an incubator at 25°C for 6‍ ‍d, colonies of *P. pinophilum* EU0013 growing from root segments were counted, and the colonization frequency was calculated as %CF=(Ncol/Nt)×100, where Ncol=the number of root segments colonized by the fungus and Nt=the total number of segments of roots studied, as described by Hata and Futai (1995).

### Total soluble sugars

Total soluble sugars were assessed using a brix refractometer (PAL-BX/ACID F5 Master Kit; ATAGO) according to the method of Tefera *et al.* (2007). Freshly harvested tomato fruits were diced into smaller pieces. Approximately 2‍ ‍g of each diced fruit was placed into tea bags and the juice was extracted by squeezing the tea bag. Two drops of the juice were placed on the prism and the measurement time was within 3 s. Distilled water was used to clean between readings and values were shown as percentages.

### Measurement of Zn, Fe, and Mn

Dried fruit samples were ignited in an electric furnace (ADVANTEC FUL220FA) at 550°C for 6‍ ‍h (heat-up time of 20‍ ‍min) and then digested in 0.6‍ ‍mol‍ ‍L^–1^ HCl. Element concentrations in solutions were assessed by atomic absorption spectrophotometry (Shimadzu AA-6600F).

### Polyphenol content in fruits

Total polyphenols in fruit samples were measured via the Folin-Ciocalteu method (Amerine and Ough, 1980). Dried samples (0.3‍ ‍g) were extracted in 70% acetone and 2.5‍ ‍mL of 10-fold diluted Folin-Ciocalteu solution was added, followed by 2.0‍ ‍mL of Na_2_CO_3_ solution (75‍ ‍g‍ ‍L^–1^) after 2‍ ‍min. Chlorogenic acid was used as the standard and absorbance was measured in a spectrophotometer (U-1800 Hitachi High Tech) at 760‍ ‍nm.

### Ascorbic acid assessment

Ascorbic acid measurements were performed using the Shimadzu Prominence HPLC system (Shimadzu) according to Brause *et al.* (2003). The extraction solution was freshly prepared by dissolving 56‍ ‍g of metaphosphoric acid in 1 L of water on the day of extraction. Plastic tubes (50‍ ‍mL) were filled into racks and 35‍ ‍mL of extraction solution was added to each tube. Frozen tomato samples (5 g) were placed into tubes and an additional 10‍ ‍mL of extraction solution was added. Samples were homogenized at 200×*g* for 30‍ ‍s and then filtered through a 110-mm filter paper. The filtrate was further filtered with a 0.45-μm sterile filter connected to a syringe and collected into a 1.5-mL tube. The HPLC syringe was used to collect 0.05‍ ‍mL of the filtrate and injected into the machine to measure concentrations. During the ana­lysis, the fitted column was a Unison UK-C18 (3‍ ‍μm, 4.6×150‍ ‍mm) ODS column (Imtakt); the mobile phase was 2‍ ‍mM HClO_4_ and 100‍ ‍mM NaBH_4_, which were pumped at flow rates of 1.0‍ ‍mL‍ ‍min^–1^ and 0.5‍ ‍mL‍ ‍min^–1^, respectively; the injection volume was 20‍ ‍μL; the column temperature was maintained at 40°C; and UV detection was performed at 300‍ ‍nm.

### Measurement of lycopene and β-carotene

Pigment concentrations were measured according to the method of Nagata and Yamashita (1992). Sample pigments were extracted once with acetone-hexane (4:6) solvent. Twenty milliliters of the solvent was measured into glass tubes, and 1‍ ‍g of the frozen tomato sample was added. Samples were then homogenized for 2‍ ‍min and kept until the solvent was separated from the solute. Three milliliters of the clear solvent/supernatant was placed in a glass cuvette and absorbance (A) was measured at 663, 645, 505, and 453‍ ‍nm in a spectrophotometer (U-1800 Hitachi High Tech). Pigment concentrations (mg 100‍ ‍mL^–1^) were calculated as stated below:

Chlorophyll a = (0.999×A_663_)–(0.0989×A_645_)

Chlorophyll b = (–0.328×A_663_)+(1.77×A_645_)

Lycopene = (–0.0458×A_663_)+(0.204×A_645_)+(0.372×A_505_)–(0.0806×A_453_)

β-Carotene = (0.216×A_663_)–(1.22×A_645_)–(0.304×A_505_)+(0.452×A_453_)

### Measurement of GA_1_, GA_4_, IAA, and ABA

The extraction and quantification of GAs, IAA, and ABA were performed as described by Manzi *et al.* (2015) with some modifications (Opio *et al.*, 2020). Approximately 0.5‍ ‍g of diced and frozen tomato fruit was crushed in 20‍ ‍mL methanol (80% [v/v]) containing butylhydroxytoluene (0.1‍ ‍g‍ ‍L^–1^) and ascorbic acid (0.1‍ ‍g‍ ‍L^–1^). Samples were homogenized and all homogenates were immediately fortified with 100‍ ‍ng of the deuterated isotopes of [^2^H_2_]-GA_1_ and [^2^H_2_]-GA_4_, with 200‍ ‍ng each of the stable isotopes of phenyl-^13^C_6_ IAA (Cambridge Isotope Laboratories) and 3′,5′,5′,7′,7′,7′-hexadeuterated ABA (ABA-d6) being used as internal standards. Samples were vortexed and stored at 4°C overnight before use. Homogenates were centrifuged the following day at 19,000×*g* at 4°C for 15‍ ‍min before filtering through a membrane filter (pore size 0.22–0.4‍ ‍μm) and then through a Sep-Pak cartridge. Filtrates were rotary-evaporated at 40°C to dryness. The aqueous layer was recovered, evaporated to dryness, dissolved in 2‍ ‍mL of 1% (v/v) acetic acid, and the solution was purified using Sep-Pak C18 cartridges (Waters). Cartridges were initially conditioned using 5‍ ‍mL of methanol in 1% acetic acid, and 5‍ ‍mL of 1% acetic acid was eluted through and discarded before samples were introduced. Once more, 5‍ ‍mL of 1% acetic acid was added with all the eluates discarded, and the solution containing phytohormones was eluted using 5‍ ‍mL of 80% methanol containing 1% acetic acid, with the eluates being collected and evaporated to dryness. The residue was then dissolved with 1‍ ‍mL of 1 M formic acid. Samples were loaded into an Oasis MCX column (60‍ ‍mg sorbent; Waters), which had been pre-conditioned with 5‍ ‍mL methanol and equilibrated with 5‍ ‍mL formic acid. The column was washed with 5‍ ‍mL formic acid with the eluent discarded. Phytohormones were subsequently eluted through the column with 5‍ ‍mL methanol (100%) and collected for purification and quantification. The quantification of GAs was performed using a LC/MS-2010EV (Shimadzu) with a cooled autosampler and LC-10ADvp pump (Shimadzu) at a voltage of 1.5 kV and column temperature of 40°C connected to a mass spectrometer equipped with an electron spray ionization (ESI) source operated in the positive analytical mode. Separation was achieved by an ODS Mightysil RP-18 column (150×2.0‍ ‍mm i.d., 5‍ ‍μm), and data acquisition software was Labsolutions Ver. 3. Using an injection volume of 1‍ ‍μL, compounds were isocratically eluted with methanol containing 20‍ ‍mM formic acid (80:20‍ ‍[v/v]) at a flow rate of 0.3‍ ‍mL‍ ‍min^–1^. LC-MS conditions were as follows: 4.5 kV for the ESI spray voltage, 250°C for the curved desolvation line (CDL) and block heater temperatures, and 1.5‍ ‍mL‍ ‍min^–1^ for the nebulizer gas (N_2_) flow. The quantification of phytohormones was performed using selected ion monitoring. The parent and fragment ions used for quantification were as follows: m/z=347 and 349 for GA_1_ and 331 and 333 for GA_4_, respectively (Opio *et al.*, 2020). The column temperature was a step gradient of 60°C for 2‍ ‍min, 60–270°C at 10°C min^–1^, and 270°C for 35‍ ‍min. The mass-to-charge ratios (m/z) were 130 and 189 for methyl-IAA, 135 and 195 for methyl-[^13^C_6_] IAA, 162 and 190 for methyl-ABA-d_0_, and 169 and 194 for methyl-[ABA-d_6_]. Endogenous IAA and ABA concentrations were calculated from the peak ratios of m/z 130/135 and 190/194, respectively (Opio *et al.*, 2020).

### Statistical ana­lysis

Data were subjected to a two-way ana­lysis of variance (ANOVA) using STATCEL version 4 (OMS) with significance set at *P*<0.05 followed by the Tukey-Kramer test for mean separation. Regarding root colonization, EU0013– groups were devoid of penicillium colonies and were excluded from the statistical ana­lysis; therefore, a one-way ANOVA was performed for the five cultivars in the EU0013+series.

## Results

### Fruit biomass, leaf chlo­rophyll content, and root colonization

**Fig. 1.**
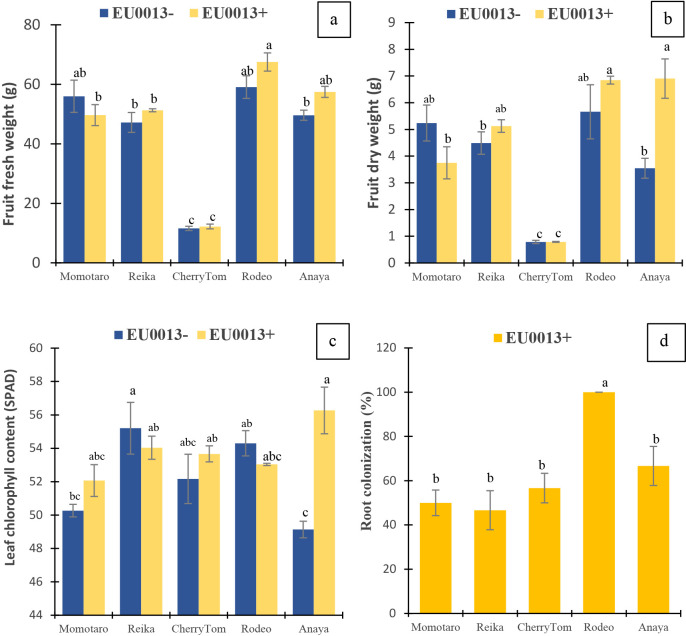
Effects of the *Penicillium pinophilum* EU0013 inoculation on (a) fruit fresh weights, (b) fruit dry weights, (c) leaf chlo­rophyll contents, and (d) root colonization in tomato cultivars. Values are means±SEM (*n*=3). ^abc^ indicate differences based on the Tukey-Kramer test.

Fruit fresh weight (Fig. 1a) showed significant differences (Table S1) due to the cultivar, with the highest values being observed in Rodeo and the lowest in Cherry. Fruit dry weight (Fig. 1b) showed significant differences due to the cultivar, inoculation, and cultivar×inoculation interaction. In Anaya, the *P. pinophilum* inoculation significantly increased the fruit dry weight over that of the uninoculated control. The leaf chlo­rophyll content (Fig. 1c) showed significant differences due to the cultivar, inoculation, and cultivar×inoculation interaction. In Anaya, the *P. pinophilum* inoculation significantly increased the leaf chlo­rophyll content over that of the uninoculated control. Root colonization by *P. pinophilum* (Fig. 1d) was significantly higher in Rodeo than in the other cultivars. Uninoculated plants were devoid of *P. pinophilum* colonies.

### Pigmentation indices

**Fig. 2.**
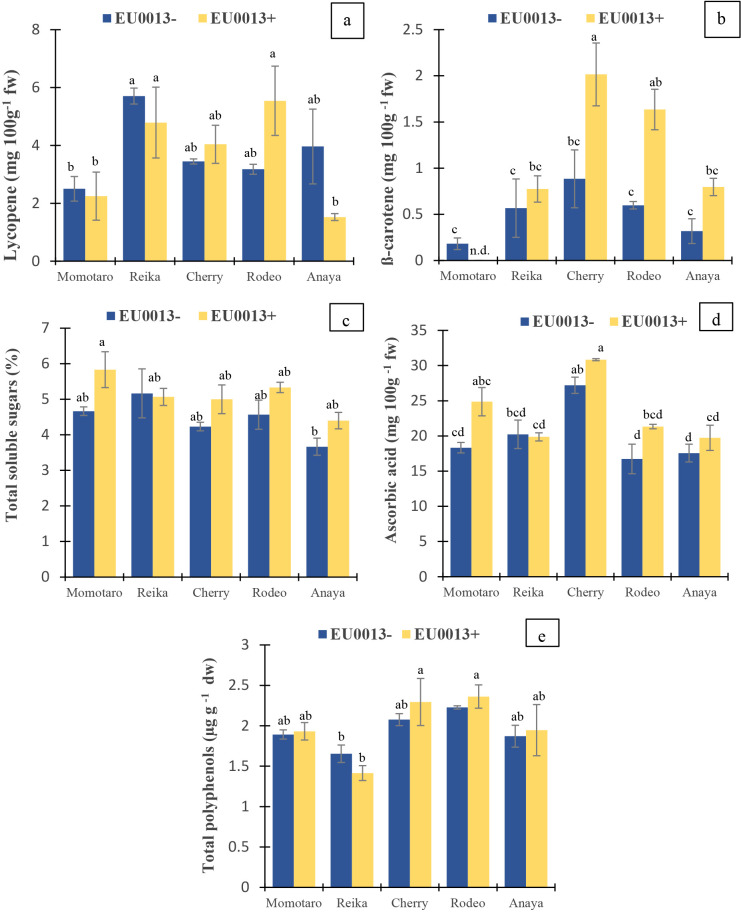
Effects of the *Penicillium pinophilum* EU0013 inoculation on fruit biochemical indices in tomato cultivars. (a) Lycopene, (b) β-carotene, (c) total soluble sugars, (d) ascorbic acid, and (e) total polyphenols. Values are means±SEM (*n*=3). ^abc^ indicate differences based on the Tukey-Kramer test. n.d.=not determined.

Lycopene (Fig. 2a) showed significant differences due to the cultivar, with the highest values being observed in Reika and Rodeo inoculated with *P. pinophilum*; however, the effect of inoculation was not significant (Table S1). β-carotene (Fig. 2b) showed significant effects of the cultivar, inoculation, and cultivar×inoculation interaction. The highest values were observed in Cherry inoculated with *P. pinophilum*, while the lowest were in Momotaro inoculated with *P. pinophilum*. In four out of five cultivars, the inoculation with *P. pinophilum* increased the β-carotene content over that of the uninoculated control.

### Total soluble sugars, ascorbic acid, and polyphenol

Total soluble sugars (Fig. 2c) showed significant differences due to the cultivar and inoculation. Except for Reika, the *P. pinophilum* inoculation increased soluble sugars in four cultivars over those in the uninoculated control. Ascorbic acid (Fig. 2d) also showed significant differences due to the cultivar and inoculation, with the highest values being observed in Cherry. Total polyphenols (Fig. 2e) showed significant differences due to the cultivar and cultivar×inoculation interaction, with the highest values being detected in Cherry and Rodeo and the lowest in Reika.

### IAA, ABA, and GAs

GA_1_ (Table 1) showed significant differences due to the cultivar×inoculation interaction. The *P. pinophilum* inoculation increased GA_1_ levels in Reika and Anaya, but decreased those in Momotaro, Cherry, and Rodeo. GA_4_ (Table 1) showed differences due to the cultivar, inoculation, and cultivar×inoculation interaction. Reika and Anaya also showed increases in GA_4_ levels due to the *P. pinophilum* inoculation, while the reverse was observed in Momotaro, Cherry, and Rodeo. IAA (Table 1) showed significant differences due to the cultivar, inoculation, and cultivar×inoculation interaction. The highest values were observed in Momotaro inoculated with *P. pinophilum*; however, values in Cherry, Rodeo, and Anaya with the *P. pinophilum* inoculation were lower than that in the uninoculated control. ABA (Table 1) showed significant differences due to the cultivar and cultivar×inoculation interaction. The *P. pinophilum* inoculation increased ABA levels in Reika and Anaya, but reduced them in Momotaro. In Reika and Anaya, the *P. pinophilum* inoculation consistently increased ABA, GA_1_, and GA_4_ levels over those in the uninoculated control.

### Zn, Fe, and Mn concentrations

Mn concentrations (Table 2) showed significant differences due to the cultivar and cultivar×inoculation interaction. The *P. pinophilum* inoculation increased Mn concentrations in Cherry, but decreased them in Momotaro, Rodeo, and Anaya. Zn concentrations (Table 2) showed significant differences due to the cultivar, with higher values being observed in Cherry. Fe concentrations (Table 2) showed significant differences due to the cultivar, inoculation, and cultivar×inoculation interaction. The *P. pinophilum* inoculation increased Fe concentrations in Reika and Cherry over that in the uninoculated control.

## Discussion

Tomato fruit development is very sensitive to environmental conditions and is strongly influenced by hormones, such as IAA and GAs (de Jong *et al.*, 2009). IAA and GAs stimulate and regulate each other during the early stages of fruit development (Koshioka *et al.*, 1994; Pattison and Catala, 2012) and are modulated by ethylene and ABA responses (Nitsch *et al.*, 2009). Furthermore, the accumulation of soluble sugars and acids in tomato fruits influence their sweetness or sourness and often depend on the ripening stage (Viskelis *et al.*, 2015). Therefore, an evaluation of endophyte effects on these fruits requires the assessment of some of these phytochemicals because of their impact on color, taste, and flavor. The *P. pinophilum* inoculation increased GA_1_, GA_4_, and ABA levels in Reika and Anaya, but reduced them in Cherry and Rodeo. This result indicates cultivar-based differences in the modulation of these phytochemicals by the endophyte against the backdrop of their physiological differences in development and responses to environmental conditions. Endophyte effects may be stimulated from the modulated expression of associated genes and their regulators (Pattison and Catala, 2012). Except for Momotaro, the *P. pinophilum* inoculation did not enhance IAA levels in the fruits. Crosstalk signaling between GAs and IAA may have minimized concurrent fluctuations in IAA levels due to the endophyte, but this was not observed in ABA (Quinet *et al.*, 2019). Lycopene and β-carotene are important pigments in tomato and play crucial roles as dietary antioxidants (Ilahy *et al.*, 2009). Their amounts are influenced by the environmental conditions of the growth, cultivar, and ripening stage of tomato (Viskelis *et al.*, 2015). Therefore, increases in β-carotene due to the *P. pinophilum* inoculation in three (Cherry, Rodeo and Anaya) out of five cultivars are remarkable. Increases were observed in ascorbic acids and total soluble sugars in four out of five cultivars, indicating the significant effects of the inoculation. These increases were not associated with the countries from which the cultivars were obtained, but rather to individual cultivar identities. Ascorbic acid is a major vitamin that enriches the human diet, scavenges free radicals, and fights against oxidative stress. Factors such as growth conditions, the cultivar, and ripening stages affect their accumulation in tomato (Viskelis *et al.*, 2015). The ascorbic acid biosynthesis pathway in plants involves D-glucose-6-P, fructose-6-P, and galactose (Hemavathi *et al.*, 2010), and these are among the main soluble sugars found in tomato (Zhao *et al.*, 2016). Therefore, similarities in the results obtained on total soluble sugars and ascorbic acid concentrations (Fig. 2c and d) are indicative of this relationship. Cherry had significantly smaller fruit weights due to its genotype/physiology. The significant effect of the *P. pinophilum* inoculation on fruit dry weights in Anaya indicates the influence of the endophyte on the accumulation of the dry biomass. This corresponds to higher leaf chlo­rophyll levels in Anaya inoculated with *P. pinophilum* and ties into the effects on sugars and ripening because the levels of total soluble sugars and dry matter in fruits slightly increase with the advancement of ripening (Viskelis *et al.*, 2015). Phytochemicals are largely linked to the phenylpropanoid pathway/polyphenol metabolism in plants (Dong and Lin, 2020). Since many plant antioxidants are phenol derivatives, the minimal impact of the inoculation on total polyphenols indicates a more targeted physiological effect of microbe-host symbiosis rather than a broad-based increase in the biosynthesis of total polyphenols given the absence of a pathogen or abiotic stress conditions (Dong and Lin, 2020; Ibiang *et al.*, 2021). Zn, Fe, and Mn are essential trace elements with important roles in the activities of many enzymes (Pilon *et al.*, 2009). Fe is essential for oxygen transport in red blood cells, Mn is involved in bone formation and some antioxidative activity, such as the healing of wounds, and Zn boosts the immune system and aids taste and smell. Since they may be deficient (*e.g.*, Fe) in the staples consumed in some communities (Okwuonu *et al.*, 2021), their natural enrichment in vegetables is obviously beneficial. Fruit Mn was increased by the *P. pinophilum* inoculation in Cherry only, while fruit Fe increased in Cherry and Reika. Enhancements in the accumulation of trace elements in tomato fruits may be achieved by the fungal modulation of root-to-shoot translocation and/or siderophore and organic acid production in the rhizosphere for improved element availability in soil, as previously reported (Khan *et al.*, 2009; Ibiang *et al.*, 2020). Significantly higher endophyte root colonization was observed in Rodeo than in the other cultivars; however, this did not clearly appear to be linked to fruit biomass. Higher root colonization by *Penicillium* may be due to signals that occur in the rhizosphere between the host and endophyte under different cultivation conditions (Ibiang *et al.*, 2021) and indicates greater compatibility between the host and endophyte.

In conclusion, *P. pinophilum* EU0013 modulated phytohormones and increased total soluble sugars, ascorbic acids, β-carotene, Mn, and Fe concentrations in tomato fruits. Its potential to enhance the nutraceutical value of tomato is indicated, particularly in Cherry and Rodeo. Cultivar responses to the *P. pinophilum* inoculation are an important factor to consider in its deployment in tomato, and responses were related to individual cultivar identities rather than the countries from which they were obtained.

## Citation

Ibiang, S. R., and Sakamoto, K. (2022) Modulation of Phytochemicals and Essential Trace Elements in Fruits of Different Tomato Cultivars by the Endophytic Fungus *Penicillium pinophilum* EU0013. *Microbes Environ ***37**: ME22026.

https://doi.org/10.1264/jsme2.ME22026

## Supplementary Material

Supplementary Material

## Figures and Tables

**Table 1.** Effects of the *Penicillium pinophilum* EU0013 inoculation on gibberellic acid, IAA, and ABA contents in fruits of tomato cultivars

		Momotaro			Reika			Cherry			Rodeo			Anaya	
		EU0013–	EU0013+		EU0013–	EU0013+		EU0013–	EU0013+		EU0013–	EU0013+		EU0013–	EU0013+
1	GA_1_ (μmol kg^–1^ fw)	87.06ab ±4.55	66.98ab ±2.65		69.91ab ±17.55	126.87ab ±34.13		177.05a ±34.02	100.73ab ±37.03		138.17ab ±46.51	67.17ab ±9.63		42.16b ±11.52	184.97a ±26.57
2	GA_4_ (μmol kg^–1^ fw)	221.02bc ±46.02	127.02c ±32.46		52.02c ±10.99	697.15a ±142.36		368.70bc ±71.36	125.07c ±14.64		520.55ab ±25.73	103.27c ±42.51		54.03c ±9.11	799.05a ±98.62
3	IAA (μmol kg^–1^ fw)	3.51b ±1.31	130.52a ±30.02		2.23b ±0.81	2.80b ±0.70		2.90b ±1.34	0.91b ±0.24		7.54b ±1.89	1.43b ±0.33		13.90b ±4.89	3.98b ±1.67
4	ABA (μmol kg^–1^ fw)	2.03ab ±0.79	0.81ab ±0.40		1.49a ±0.63	3.96a ±1.10		1.93ab ±1.60	0.57ab ±0.05		0.29b ±0.12	0.36b ±0.14		1.30ab ±0.76	3.98a ±0.13

Values are means±SEM (*n*=3). ^abc^ indicate differences based on the Tukey-Kramer test.

**Table 2.** Effects of the *Penicillium pinophilum* EU0013 inoculation on Zn, Fe, and Mn concentrations in fruits of tomato cultivars.

		Momotaro			Reika			Cherry			Rodeo			Anaya	
		EU0013–	EU0013+		EU0013–	EU0013+		EU0013–	EU0013+		EU0013–	EU0013+		EU0013–	EU0013+
1	Zn (μg g^–1^)	27.99ab ±2.34	27.19ab ±2.37		25.92ab ±1.41	28.72ab ±1.74		37.28a ±6.90	32.03a ±1.84		25.86b ±1.15	24.89b ±0.72		28.65ab ±0.78	30.62ab ±2.35
2	Fe (μg g^–1^)	53.87b ±7.24	45.08b ±6.96		62.47ab ±7.29	107.78a ±14.23		52.85b ±11.25	90.45ab ±10.88		77.96ab ±6.78	76.84ab ±11.20		62.21ab ±10.67	66.24ab ±11.78
3	Mn (μg g^–1^)	20.93b ±5.27	11.45bc ±1.55		11.60bc ±3.26	14.72bc ±1.72		12.38bc ±1.95	36.33a ±2.71		16.11bc ±1.90	9.16c ±1.08		23.77ab ±0.94	15.51bc ±3.02

Values are means±SEM (*n*=3). ^abc^ indicate differences based on the Tukey-Kramer test.
