# Supplementary material for: Modulation of Phytochemicals and Essential Trace Elements in Fruits of Different Tomato Cultivars by the Endophytic Fungus Penicillium pinophilum EU0013
Source: Microbes Environ. 2022 Sep 14;37(3):ME22026. doi: 10.1264/jsme2.ME22026 (PMC9530726; doi:10.1264/jsme2.ME22026)
Supplement: Supplementary file 1 — Supplementary Material [file 37_22026_s1.pdf]

Table S1. *P* values showing significance of treatment effects and their interaction based on ANOVA

	Parameter	Cultivar	Inoculation	Cultivar × Inoculation
1	Lycopene	0.008**	0.794 ns	0.064 ns
2	β-carotene	1.96E-05***	0.00049***	0.0204*
3	GA_1	0.259 ns	0.705 ns	0.002**
4	GA_4	0.0065**	0.0052**	1.05E-08***
5	IAA	5.69E-06***	0.001**	1.68E-06***
6	ABA	0.259 ns	0.38 ns	0.047*
7	Soluble sugars	0.020*	0.007**	0.514 ns
8	Ascorbic acids	1.69E-06***	0.0013**	0.185 ns
9	Polyphenols	0.0015**	0.6614 ns	0.6913 ns
10	Penicillium root colonization	0.0016 **		
11	Leaf chlorophyll	0.023*	0.015*	0.001**
12	Fruit fresh wt.	1.23E-12***	0.126 ns	0.109 ns
13	Fruit dry wt.	2.49E-08***	0.038*	0.003**
14	Mn	0.001**	0.779	1.27E-05***
15	Zn	0.03*	0.797 ns	0.625 ns
16	Fe	0.022*	0.0259*	0.048*

*=P<0.05; **=P<0.01; ***=P<0.001; ns=not significant. One-way ANOVA (Penicillium root colonization), Two-way ANOVA (other parameters).
